# Assessment of Reproductive Health Knowledge Among College Students in Northwestern India: A Cross-Sectional Study

**DOI:** 10.7759/cureus.54681

**Published:** 2024-02-22

**Authors:** Shilpa Dutta, Akash More, Sanket Mahajan, Neha Nawale, Namrata Choudhary, Deepti Shrivastava

**Affiliations:** 1 Clinical Embryology, Datta Meghe Institute of Higher Education and Research, Wardha, IND; 2 Obstetrics and Gynaecology, Jawaharlal Nehru Medical College, Datta Meghe Institute of Higher Education and Research, Wardha, IND

**Keywords:** taboo, health, students, reproduction, knowledge, infertility

## Abstract

Background

Reproductive health knowledge is a critical aspect of overall well-being, particularly among college students who represent a demographic transitioning into adulthood. In northwestern India, where cultural nuances and societal perceptions play a significant role, understanding the factors influencing reproductive health knowledge becomes imperative. This cross-sectional study explores the interplay between demographic factors and awareness of reproductive health and infertility treatment among college students in northwestern India.

Methods

A diverse sample of 564 college students in northwestern India participated in the study, providing information on key demographic variables, including age, gender, marital status, degree, field of study, and college year. Statistical analysis, including the calculation of p-values, was employed to determine the significance of associations between these demographic factors and the participants' knowledge of reproductive health. Descriptive statistics, including percentages and numbers, were calculated to present a comprehensive overview of the data. To evaluate the significance of associations, chi-square tests were conducted for categorical variables such as age, gender, marital status, degree, field of study, and college year. The p-values were computed to determine the statistical significance of observed relationships, with a significance level set at 0.05.

Results

The study uncovered notable findings with implications for targeted interventions. Among age groups, participants aged 23-25 exhibited the highest knowledge percentage at 43.22% (51/564), and this association was statistically significant (p = 0.042). Gender disparities were evident, with females showing higher awareness (46.52% (127/564)) compared to males, and this difference was statistically significant (p = 0.001). Marital status revealed significant differences (p = 0.0012), particularly in single individuals who demonstrated a knowledge percentage of 46.52% (127/564). Significant variations were observed based on the degree held, with doctorate holders having the highest awareness at 49.15% (58/564) (p = 0.01). Field of study significantly influenced knowledge (p = 0.0001), particularly in medical and engineering disciplines. College year also exhibited significance (p = 0.003), with the first-year students demonstrating a knowledge percentage of 42.20% (73/564).

Conclusions

These findings underscore the importance of tailored educational interventions and targeted awareness campaigns. Recognizing the influence of demographic factors on reproductive health knowledge is crucial for developing effective strategies that address the specific needs of college students in northwestern India, promoting a more informed approach to reproductive health and infertility treatment.

## Introduction

World Health Organization (WHO) defines infertility as the inability to conceive after at least 12 months of regular sexual activity without contraception [1]. Infertility constitutes a significant global health challenge, affecting approximately 12-15% of couples worldwide [2]. Worldwide, overall fertility rates are declining steeply, possibly influenced by prolonged time to conceive among both men and women for their first child [3]. Current literature suggests that approximately 86% of the young population resides in developing countries, with a total of around 1.7 million young cohorts globally [4]. India hosts about 65% of this young population, according to the latest report of 2020 [5]. Therefore, acquiring proper reproductive health knowledge is imperative to avoid unforeseen fertility issues. The factors contributing to infertility and unfavorable pregnancy outcomes are multifaceted and arise from intricate interactions among genetic, environmental, and lifestyle factors prevalent in the general population [6]. It has been reported that more than one-third of India's younger population suffers from sexually transmitted diseases such as acquired immune deficiency syndrome (AIDS), indicating a lack of active awareness about reproductive health and safety measures among the younger cohort [7].

It has been observed that knowledge about fertility heavily influences decisions concerning reproductive health [3]. In 2017, the International Glossary of Infertility and Fertility Care defined fertility awareness as the understanding of reproduction and fertility, encompassing various individual risk factors such as advanced age, sexual health considerations, sexually transmitted infections, and lifestyle factors like smoking and obesity. Furthermore, it entails the recognition of non-personal risk elements, including environmental and workplace factors. This understanding extends to awareness of the social and cultural influences on family planning decisions, as well as the societal and cultural dynamics impacting the desire for family formation [3].

A subset of individuals may engage in novel behaviors such as substance abuse, unprotected sexual activity, and involvement with multiple sexual partners due to hormonal changes, depression, and peer influence [8]. There is a scarcity of research, primarily conducted abroad, concerning the knowledge base of university students regarding infertility. Despite their aspirations for parenthood, these students exhibit a heightened risk of infertility due to intensive efforts to achieve academic and career objectives [9,10]. Gavin et al. observed that fostering positive youth development programs plays a crucial role in advancing the sexual and reproductive health of adolescents and young individuals [11].

Studies have reported that university students tend to express a desire to have children as they approach a stage of declining reproductive capacity. Nevertheless, they exhibit a deficit in understanding age-related decreases in fertility, along with relevant risk factors and infertility issues [8,12,13]. Hence, this article focuses on assessing the reproductive health knowledge among college students in northwestern India and their awareness of infertility treatment. The results of this study will help evaluate further plans needed for the targeted population to improve fertility outcomes accordingly.

## Materials and methods

Study design

The study design for the article was cross-sectional. It included 600 elected participants through a snowball sampling technique. A Google form was distributed among the participants with a preset of questionnaires having 16 multiple-choice questions. Individuals were conveniently selected and were further encouraged to circulate the questionnaire survey among other participants who met the criteria of the study. Initial participants were elected through known connections, communities, and pre-existing networks. Further, participants were elected through known contacts of the initial group of selected participants, which fostered a snowball sampling method. After thorough data cleaning, 564 eligible participants’ answers from the questionnaire were selected to be evaluated for the study. The anonymity of the respondents was maintained throughout to ensure the participant's privacy, and hence, the offered responses were candid and truthful. 

Data analysis

Descriptive statistics was utilized for the quantification of the answers obtained through the Google form survey. The chi-square (χ^2^) test was used for the calculation of the statistical value. A p-value less than 0.05 was considered to be statistically significant for the analysis of the data collected. SPSS 26.0 for Windows Student Version (IBM Inc., Armonk, New York) was used for the evaluation of the study.

Ethical considerations

Prior to conducting the online questionnaire survey, informed consent was diligently obtained from all participants. This study has been ethically approved by the Institutional Review Board of Datta Meghe Institute of Higher Education and Research in its meeting held on September 2, 2023, having reference number DMIHER(DU)/IEC/2023/1321.

## Results

As shown in Table 1, the demographic data reveals a diverse distribution of participants across various age groups, genders, and marital statuses. In terms of age, the majority of respondents fall within the 23-25 age bracket, comprising 45.04% (254/564) of the sample, followed by those aged 20-22 (24.11% (136/564)) and 17-19 (8.69% (49/564)). The gender distribution shows a relatively balanced representation, with 48.05% (271/564) identified as male and 51.95% (293/564) as female. Regarding marital status, the participants are spread across single (40.78% (230/564)), married (34.93% (197/564)), and separated (24.29% (137/564)) categories. This demographic snapshot provides valuable insights into the composition of the surveyed population, allowing for a more nuanced understanding of the factors under consideration in the study.

**Table 1:** Demographic data of the participants The data has been represented as N = Total number of participants who selected the aforementioned option among all the choices available; % = N/Total number of participants selected for the study (564)

Demographic Data	Number (N)	%
Age (years)		
17-19	49	8.69 (49/564)
20-22	136	24.11 (136/564)
23-25	254	45.04 (254/564)
25-27	74	13.12 (74/564)
27-29	32	5.67 (32/564)
30+	19	3.36 (19/564)
Gender		
Male	271	48.05 (271/564)
Female	293	51.95 (293/564)
Marital Status		
Single	230	40.78 (230/564)
Married	197	34.93 (197/564)
Separated	137	24.29 (137/564)

The participant background data, as shown in Table 2, provides a comprehensive overview of the educational and academic profile of the surveyed individuals. Among the degrees held by respondents, the majority possess a Bachelor's degree (29.61% (167/564)), followed closely by those with a Master's degree (24.11% (136/564)) and Doctorate holders (21.45% (121/564)). Diploma holders constitute 14.36% (81/564), and there is a notable presence of individuals with other qualifications (10.46% (59/564)). When considering college years, the distribution is fairly even, with the highest representation in the first year (28.72% (162/564)) and descending proportions in subsequent years. The field of study diversity is evident, with Commerce (18.44% (104/564)), Arts (12.41% (70/564)), and Law (14.01% (79/564)) being the most prevalent disciplines. This comprehensive breakdown of participant backgrounds illuminates the varied educational journeys and fields of expertise within the surveyed group, providing valuable context for interpreting responses and drawing insights into the research study.

**Table 2:** Background of the participants The data has been represented as N = Total number of participants who selected the aforementioned option among all the choices available; % = N/Total number of participants elected for the study (564)

Participant Background	Number (N)	%
Degree		
Diploma	81	14.36 (81/564)
Bachelor's	167	29.61 (167/564)
Master's	136	24.11 (136/564)
Doctorate	121	21.45 (121/564)
Others	59	10.46 (59/564)
College Year		
First	162	28.72 (162/564)
Second	97	17.19 (97/564)
Third	136	24.11 (136/564)
Fourth	91	16.13 (91/564)
Fifth	78	13.83 (78/564)
Field of Study		
Medical	48	8.51 (48/564)
Engineering	49	8.69 (49/564)
Arts	70	12.41 (70/564)
Commerce	104	18.44 (104/564)
Science	40	7.09 (40/564)
Law	79	14.01 (79/564)
Humanities	86	15.25 (86/564)
Management	68	12.05 (68/564)
Others	20	3.55 (20/564)

The data on reproductive health awareness, as shown in Table 3, sheds light on participants' attitudes, knowledge, and confidence regarding this critical aspect of well-being. A significant portion of respondents actively seek information about their reproductive health, with 52.66% (297/564) indicating that they do so sometimes and 18.62% (105/564) doing it very often. In contrast, 28.72% (162/564) of participants never seek such information. The distribution of previous knowledge about reproductive health and infertility treatment is varied, with 48.40% (273/564) being unsure, 30.67% (173/564) having no previous knowledge, and 20.92% (118/564) affirming their awareness. Confidence levels in the accuracy of reproductive health information vary as well, with 40.60% (229/564) expressing a lack of confidence and 22.16% (125/564) feeling somewhat confident. The most common sources for seeking information are friends (48.76% (275/564)) and the internet (24.11% (136/564)), showcasing the role of social networks and online platforms in disseminating reproductive health knowledge. This comprehensive data provides insights into the participants' engagement with and trust in reproductive health information, highlighting areas for targeted awareness campaigns and education.

**Table 3:** Reproductive health awareness among the participants The data has been represented as N = Total number of participants who selected the aforementioned option among all the choices available; % = N/Total number of participants elected for the study (564)

Reproductive Health Awareness	Number (N)	%
Do you seek information regarding your reproductive health?		
Very often	105	18.62 (105/564)
Sometimes	297	52.66 (297/564)
Never	162	28.72 (162/564)
Do you have previous knowledge about reproductive health and infertility treatment?		
Yes	118	20.92 (118/564)
No	173	30.67 (173/564)
Not sure	273	48.40 (273/564)
How confident are you in the accuracy of the reproductive health information you come across?		
Very confident	94	16.67 (94/564)
Somewhat confident	125	22.16 (125/564)
Neutral	116	20.57 (116/564)
Not confident	229	40.60 (229/564)
Where do you usually seek information about reproductive health and fertility?		
Friends	275	48.76 (275/564)
Family	71	12.59 (71/564)
Internet	136	24.11 (136/564)
Healthcare professionals	24	4.25 (24/564)
Others	58	10.28 (58/564)

The social outlook data, as shown in Table 4, provides valuable insights into the perceptions and attitudes of participants towards reproductive health in the broader societal context. A substantial majority, comprising 50.35% (284/564), strongly agree that there is societal pressure associated with the ability to reproduce, and an additional 28.72% (162/564) agree with this sentiment. Conversely, only a small percentage, 7.97% (45/564), disagrees with this notion, emphasizing the prevalence of societal expectations regarding fertility. A significant portion, 62.94% (355/564), believe that there is a stigma attached to discussing or seeking help for infertility, while 14.54% (82/564) are unsure. Assessing their knowledge about factors influencing infertility and methods to conceive, participants express a range of confidence levels, with 21.63% (122/564) rating their knowledge as very good and 15.43% (87/564) as very poor. This data underscores the importance of understanding societal perceptions and stigma surrounding reproductive health, advocating for more open conversations, and addressing knowledge gaps to foster a supportive and informed community.

**Table 4:** Social outlook of the participants The data has been represented as N = Total number of participants who selected the aforementioned option among all the choices available; % = N/Total number of participants elected for the study (564)

Social Outlook	Number (N)	%
Do you believe it is not perceived well by society if someone is not able to reproduce?		
Strongly agree	284	50.35 (284/564)
Agree	162	28.72 (162/564)
Neither agree or disagree	70	12.41 (70/564)
Disagree	45	7.97 (45/564)
Strongly disagree	3	0.53 (3/564)
In your opinion, is there a stigma associated with discussing or seeking help for infertility?		
Yes	355	62.94 (355/564)
No	127	22.52 (127/564)
Not sure	82	14.54 (82/564)
How would you rate your knowledge about the factors influencing infertility and methods to conceive?		
Very Poor	87	15.43 (87/564)
Poor	104	18.44 (104/564)
Neutral	180	31.92 (180/564)
Good	71	12.59 (71/564)
Very Good	122	21.63 (122/564)

The data on infertility awareness, as shown in Table 5, reflects a spectrum of knowledge and perceptions among participants. A significant portion, comprising 24.82% (140/564), is aware of factors contributing to infertility, while 31.56% (178/564) are not and 43.62% (246/564) are unsure. Interestingly, when asked to enumerate factors contributing to infertility, participants demonstrated varied levels of awareness. Notable factors such as age, lifestyle choices, and medical conditions were well-recognized, with 94.15% (531/564) identifying sexually transmitted infections (STIs) like AIDS and 90.78% (512/564) associating smoking, excessive alcohol consumption, and drug use with infertility. However, unconventional factors like weather, tight underwear, and laptop use also garnered responses. About 66.49% (375/564) of participants believe that infertility can be treated, while 18.08% (102/564) think otherwise and 15.43% (87/564) are unsure. This diversity in responses underscores the need for targeted education to enhance public awareness about infertility causes and treatments, fostering informed perspectives on this significant reproductive health issue.

**Table 5:** Infertility awareness among the participants The data has been represented as N = Total number of participants who selected the aforementioned option among all the choices available; % = N/Total number of participants elected for the study (564)

Infertility Awareness	Number (N)	%
Are you aware of factors that can contribute to infertility?		
Yes	140	24.82 (140/564)
No	178	31.56 (178/564)
Maybe	246	43.62 (246/564)
Can you enlist all factors that can contribute to infertility by selecting from the below options?		
Age	487	86.35 (487/564)
Smoking, excessive alcohol consumption, drug use	512	90.78 (512/564)
Weight gain/loss	375	66.49 (375/564)
Stress	278	49.29 (278/564)
Sexually transmitted infections (STIs) like AIDS	531	94.15 (531/564)
Poor diet	364	64.54 (364/564)
Weather	201	35.64 (201/564)
Medical Conditions: Conditions like diabetes, thyroid disorders, etc	215	38.12 (215/564)
Hormonal Imbalance	97	17.20 (97/564)
Genetic Factors	82	14.54 (82/564)
Reproductive organ Issues	112	19.86 (112/564)
Medications	107	18.97 (107/564)
Previous Surgeries	71	12.59 (71/564)
Tight Underwear	136	24.11 (136/564)
Laptop Use	46	8.16 (46/564)
Cycling	30	5.32 (30/564)
Cell Phone Radiation	89	15.78 (89/564)
Birth Control Pill Use	53	9.39 (53/564)
Masturbation	78	13.83 (78/564)
Breastfeeding	15	2.66 (15/564)
All of above	249	44.15 (249/564)
Not sure of options given	278	49.29 (278/564)
Do you think infertility can be treated?		
Yes	375	66.49 (375/564)
No	102	18.08 (102/564)
Not sure	87	15.43 (87/564)

The presented data, as shown in Table 6, illustrates the distribution of participants' responses based on their knowledge of reproductive health and infertility treatment, segmented by various factors. The p-values associated with each factor indicate the statistical significance of the observed differences, with a p-value less than 0.05 considered as statistically significant. In terms of age, respondents aged 23-25 exhibited the highest awareness, with a p-value of 0.042. Gender significantly influenced knowledge, as males displayed higher awareness compared to females (p = 0.001). Marital status also played a significant role, with single individuals having greater knowledge than their married or separated counterparts (p = 0.0012). The educational background showed a noteworthy impact, with participants holding doctorate degrees being the most informed group (p = 0.01). Field of study emerged as a crucial factor, with medical and engineering students exhibiting higher awareness compared to other disciplines (p = 0.0001). Additionally, college years demonstrated a statistically significant effect on knowledge, with fifth-year students being the most informed (p = 0.003). These findings underscore the importance of considering demographic variables in understanding and addressing awareness levels regarding reproductive health and infertility treatment.

**Table 6:** Factors associated with participants' knowledge about reproductive health and infertility treatment The data has been represented as N = Total number of participants who selected the aforementioned option among all the choices available; % = N/Total number of participants elected for the study (564) P-value < 0.05 is considered as statistically significant in the study

Factors	Do you have previous knowledge about reproductive health and infertility treatment?						P-Value
	Yes		Not Sure		No		
	Number (N)	%	Number (N)	%	Number (N)	%	
Age (years)							0.042
17-19	6	5.08 (6/564)	17	6.23 (17/564)	26	15.03 (26/564)	
20-22	21	17.79 (21/564)	64	23.44 (64/564)	51	29.48 (51/564)	
23-25	51	43.22 (51/564)	136	49.82 (136/564)	67	38.73 (67/564)	
25-27	21	17.79 (21/564)	35	12.82 (35/564)	18	10.40 (18/564)	
27-29	8	6.78 (8/564)	15	5.49 (15/564)	9	5.20 (9/564)	
30+	11	9.32 (11/564)	6	2.19 (6/564)	2	1.16 (2/564)	
Gender							0.001
Male	71	60.17 (71/564)	146	53.48 (146/564)	54	31.21 (54/564)	
Female	47	39.83 (47/564)	127	46.52 (127/564)	119	68.79 (119/564)	
Marital Status							0.0012
Single	21	17.79 (21/564)	127	46.52 (127/564)	82	47.40 (82/564)	
Married	52	44.06 (52/564)	78	28.57 (78/564)	67	38.73 (67/564)	
Separated	45	38.14 (45/564)	68	24.91 (68/564)	24	13.87 (24/564)	
Degree							0.01
Diploma	11	9.32 (11/564)	48	17.58 (48/564)	22	12.72 (22/564)	
Bachelor's	17	14.40 (17/564)	86	31.50 (86/564)	64	36.99 (64/564)	
Master's	24	20.34 (24/564)	67	24.54 (67/564)	45	26.01 (45/564)	
Doctorate	58	49.15 (58/564)	44	16.12 (44/564)	19	10.98 (19/564)	
Others	8	6.78 (8/564)	28	10.26 (28/564)	23	13.29 (23/564)	
Field of Study							0.0001
Medical	36	30.50847	10	3.66 (10/564)	2	1.16 (2/564)	
Engineering	12	10.16949	28	10.26 (28/564)	9	5.20 (9/564)	
Arts	11	9.322034	35	12.82 (35/564)	24	13.87 (24/564)	
Commerce	8	6.779661	57	20.88 (57/564)	39	22.54 (39/564)	
Science	19	16.10169	13	4.76 (13/564)	8	4.62 (8/564)	
Law	17	14.40678	46	16.85 (46/564)	16	9.25 (16/564)	
Humanities	5	4.237288	36	13.19 (36/564)	45	26.01 (45/564)	
Management	3	2.542373	40	14.65 (40/564)	25	14.45 (25/564)	
Others	7	5.932203	8	2.93 (8/564)	5	2.89 (5/564)	
College Year							0.003
First	22	18.64407	67	24.54 (67/564)	73	42.20 (73/564)	
Second	15	12.71186	53	19.41 (53/564)	29	16.76 (29/564)	
Third	17	14.40678	82	30.03 (82/564)	37	21.39 (37/564)	
Fourth	28	23.72881	42	15.38 (42/564)	21	12.14 (21/564)	
Fifth	36	30.50847	29	10.62 (29/564)	13	7.52 (13/564)	

## Discussion

The findings from this study of the cross-sectional data set aimed at assessing reproductive health awareness among northwestern Indian college students are profound. It has been observed by researchers that due to insufficient knowledge about reproductive health, college students tend to make less use of existing reproductive healthcare facilities available [14]. Thus, this leads to an increasing trend of reproductive health problems among the reproductive age cohorts. Studies from Kenya, China, Kerala, Turkey, and other countries have reportedly evaluated and pointed out the fact that there is a substantial lack of reproductive health knowledge among different sociodemographic profiles, and the percentage of the population that contributes majorly belongs to the college students [4,15-18].

Gender-wise distribution of reproductive knowledge appears to be balanced; however, upon a closer analysis of the respondents’ data, it is observed that males had a slight upper hand as compared to females in reproductive health knowledge. This finding can be attributed to the fact that males have comparatively easier access to available information about reproductive health awareness or social expectations. Studies from India are scarce, and from the limited research literature available, it has been reported that females tend to generally have poor knowledge of sexual health, leading to several fertility-related issues [19,20]. Upon analyzing the answers from an educational background perspective, it has been observed that individuals holding degrees in medical, engineering, and higher educational qualifications, such as doctorates, tend to have better reproductive knowledge as compared to other qualifications. Both developed, and developing countries have a trend of high-risk sexual behaviors predominantly among the younger age cohorts, thereby increasing incidents of unwanted pregnancies due to a lack of safer intercourse knowledge [21]. Hence, it is a matter of utmost importance to spread awareness about reproductive health among college students to curb the incidence of unwanted pregnancies. Parents hold the primary role as the initial educators of the young population regarding these topics, influencing the development of adolescents' attitudes and behaviors toward sexuality significantly [21]. However, many studies have reported that young adults tend to seek reproductive health knowledge from social sites, friends, and unreliable sources, while parents and siblings are the least used knowledge sources [22]. These generally lead to incorrect sharing of information among the reproductive groups, thereby increasing stances of high-risk sexual behaviors, leading to STIs and infertility.

Although the majority of college students have shown intention in many literatures to postpone childbearing, their understanding of age-related fertility decline was found to be limited, and they tended to overestimate the success rates of fertility treatments [23]. Previous studies from the United States and Sweden have highlighted that individuals having college degrees tend to have their first child above the age of 30 years, which is much higher than those having lesser qualifications [24,25]. However, such studies in India are limited, which is why this study tried to bridge the knowledge gap. Our study was in line with other research around the world that the younger age cohort particularly lacks awareness of reproductive health and participates in unsafe intercourse, which majorly contributes to STIs in India. Thus, it is of utmost importance to build a robust awareness plan to increase the knowledge of fertility health and stances related to infertility to gain control over the increasing rate of infertility rise in India and improve the overall reproductive health of the major population group of India.

## Conclusions

Our study sheds light on the intricate relationship between demographic factors and reproductive health knowledge among college students in northwestern India. The findings underscore the significance of age, gender, marital status, educational background, field of study, and college year in influencing awareness of reproductive health and infertility treatment. Noteworthy patterns emerged, such as higher awareness among individuals aged 23-25, females, singles, doctorate holders, and students in specific fields like medical and engineering disciplines. These insights emphasize the need for targeted educational interventions and awareness campaigns tailored to the diverse characteristics of college students. Recognizing the influence of demographic factors on reproductive health knowledge is imperative for developing effective strategies that empower this population to make informed decisions about their reproductive well-being. Addressing these demographic-specific needs has the potential to enhance overall awareness and contribute to a more proactive and informed approach to reproductive health and infertility treatment among college students in northwestern India.
